# miR-125b Regulates the Early Steps of ESC Differentiation through Dies1 in a TGF-Independent Manner

**DOI:** 10.3390/ijms140713482

**Published:** 2013-06-27

**Authors:** Marica Battista, Anna Musto, Angelica Navarra, Giuseppina Minopoli, Tommaso Russo, Silvia Parisi

**Affiliations:** 1 Department of Molecular Medicine and Medical Biotechnology, University of Naples, “Federico II”, Via Sergio Pansini 5, 80131 Naples, Italy; E-Mails: battistam@ceinge.unina.it (M.B.); anna.musto@unina.it (A.M); angelica.navarra@unina.it (A.N.); minopoli@dbbm.unina.it (G.M.); tommaso.russo@unina.it (T.R.); 2 Ceinge Biotecnologie Avanzate, Via Gaetano Salvatore 486, 80145 Naples, Italy; 3 European School of Molecular Medicine (SEMM), 80145 Naples, Italy

**Keywords:** miRNAs, TGFβ, embryonic stem cells, differentiation, epiblast stem cells, signaling

## Abstract

Over the past few years, it has become evident that the distinctive pattern of miRNA expression seen in embryonic stem cells (ESCs) contributes to important signals in the choice of the cell fate. Thus, the identification of miRNAs and their targets, whose expression is linked to a specific step of differentiation, as well as the modulation of these miRNAs, may prove useful in the learning of how ESC potential is regulated. In this context, we have studied the expression profile of miRNAs during neural differentiation of ESCs. We have found that miR-125b is upregulated in the first steps of neural differentiation of ESCs. This miRNA targets the BMP4 co-receptor, Dies1, and, in turn, regulates the balance between BMP4 and Nodal/Activin signaling. The ectopic expression of miR-125b blocks ESC differentiation at the epiblast stage, and this arrest is rescued by restoring the expression of Dies1. Finally, opposite to miR-125a, whose expression is under the control of the BMP4, miR-125b is not directly regulated by Transforming Growth Factor beta (TGFβ) signals. These results highlight a new important role of miR-125b in the regulation of the transition from ESCs to the epiblast stage and add a new level of control on TGFβ signaling in ESCs.

## 1. Introduction

Embryonic stem cells (ESCs) have the unique characteristics of self-renewing in culture and generating a wide range of specialized cell types upon differentiation cues. For their characteristics, they represent an appealing system to study embryonic development and, more importantly, to obtain functional cells for replacement therapy. Of course, a precise and detailed knowledge of the gene programs regulating ESC fate is necessary to allow the use of these cells. Among the signals that regulate ESC fate, there are extrinsic signals, such as Leukemia Inhibitory Factor (LIF) and Transforming Growth Factor beta (TGFβ). The LIF pathway is already well characterized, and it is required to maintain the undifferentiated state of ESCs [1]. TGFβ pathways, *i.e.*, Bone Morphogenetic Protein 4 (BMP4) and Nodal/Activin, have multiple functions, both in stemness maintenance and during the differentiation [2–6], and they seem to work in a perfectly balanced way, so that a misregulation of one of these pathways has relevant consequences on the other ones [7,8]. Another important and widely studied class of regulators of ESC gene programs are the transcription factors (TFs), which have the important characteristic that one TF may control the expression of numerous genes to execute whole differentiation programs [9,10]. Another emerging cohort of molecules holding the same ability is that of miRNAs, the non-coding RNA assigned to regulate post-transcription gene expression. Like TFs, the temporal expression of miRNAs is highly regulated and responsive to changes, depending on the stem cell status. With the aim of deeply understanding the biology of ESCs and to learn more in the control of their potential, these cells have been profiled using different methods to identify miRNAs that have potential roles in stemness and differentiation [11,12]. These studies have revealed several miRNA families that are highly expressed, specifically, in undifferentiated cells [13], as well as the miRNAs that are specifically expressed when differentiation occurs, such as the well-known family of let-7 [14]. Over the past few years, it has become apparent that the distinctive pattern of miRNA expression seen in ESCs contributes to many of the unique phenotypic properties of these cells. Indeed, the switch from pluripotent to lineage-specific cells is marked by the downregulation of pluripotency markers and the activation of lineage-specific gene expression, which are accompanied by changes in the expression of many miRNAs. Whereas some miRNAs function in promoting exit from the pluripotent state by targeting pluripotency factors, other miRNAs stabilize the pluripotent state [15–18]. The introduction or depletion of miRNAs involved in regulating ESC fate may be useful in inducing differentiation along a particular lineage. Recent discoveries have revealed a model in which miRNA regulatory events are linked with transcription factor and signaling networks that control cell fate and differentiation, modulating their activity through positive and negative feedback loops to modulate stem cell fate decisions [19–21]. We have performed a miRNA profiling in ESCs undergoing neural differentiation, and we have demonstrated that some miRNAs are able to control ESC differentiation by targeting important regulators of chromatin remodeling [22]. Furthermore, we have recently illustrated a model in which miR-125a is linked to the signaling of BMP4 and Nodal/Activin, modulating their activity through negative feedback loops to regulate ESC fate decisions [8]. Another miRNA belonging to the same family of miR-125a is miR-125b. We have analyzed the function of this miRNA, which shows an interesting expression profile during ESC differentiation. In this paper, we show that the modulation of this miRNA results in significant changes of the ESC state. These effects are due to the modulation of BMP4 and Nodal signaling during the first step of ESC differentiation. Moreover, we found that miR-125b, opposite to that observed for miR-125a, does not convey the control from TGFβ signaling.

## 2. Results and Discussion

### 2.1. miR-125b Overexpression Blocks ESC at the Epiblast Stage

**Figure 1:**
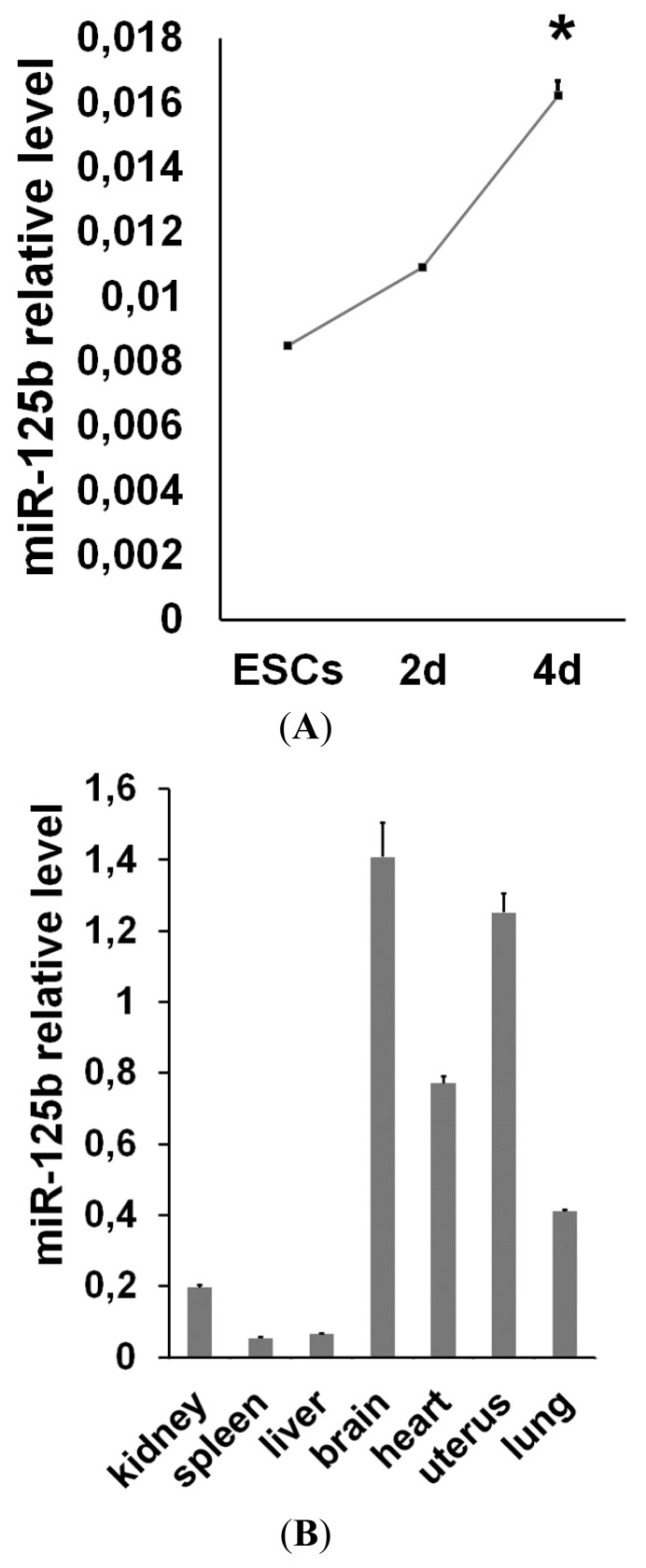
miR-125b expression in embryonic stem cells (ESCs) and mouse tissues. (**A**) miR-125b expression levels were analyzed by qPCR in undifferentiated ESCs and during neural differentiation through serum-free embryoid bodies (SFEBs) formation; (**B**) Analysis of miR-125b expression in mouse adult tissues. The data were normalized to the U6 internal control (*p < 0.05).

**Figure 2:**
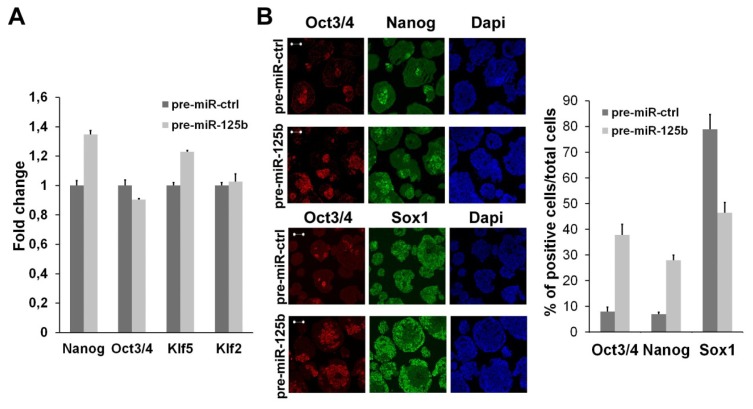

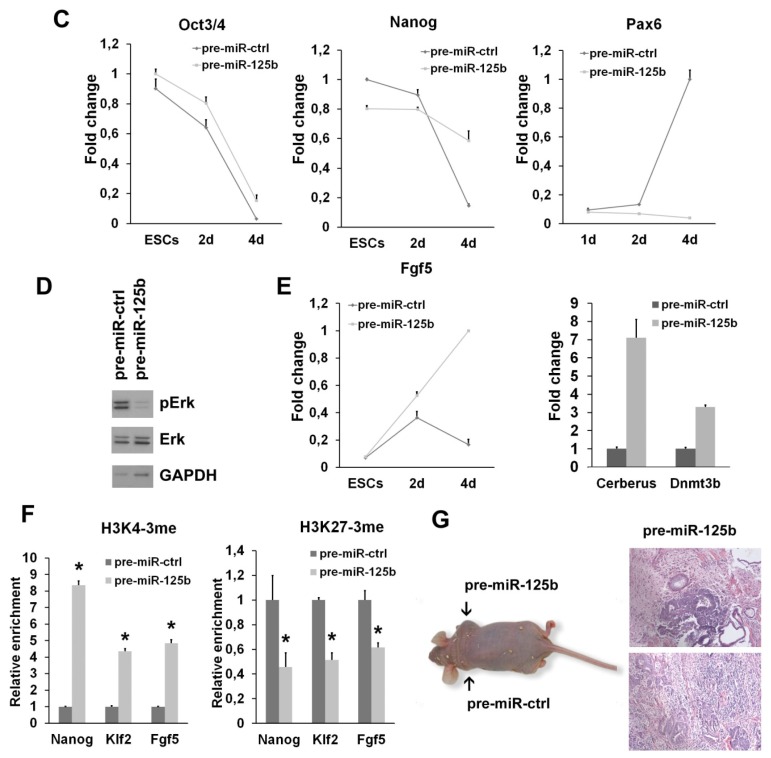
Effects of miR-125 ectopic expression *in vitro* and *in vivo*. (**A**) Analysis of stemness markers (Oct3/4, Nanog, Klf2 and Klf5) in undifferentiated ESCs transfected with pre-miR-125b or with control pre-miR (pre-miR-ctrl). The fold change is calculated by assigning the arbitrary value, one, to the amount found in cells transfected with control pre-miRNA; (**B**) Immunofluorescence analysis of four-day differentiated SFEBs upon miR-125b overexpression. Markers of pluripotency (Oct3/4, Nanog) and neuroectoderm (Sox1) are shown. Scale bar: 50 μm; (**C**) qPCR analysis of stemness (Oct3/4 and Nanog) and neuroectodermal (Pax6) markers in differentiating ESCs upon miR-125b overexpression. The fold change is calculated by assigning the arbitrary value, one, to the time point showing the highest amount of the indicated mRNA; (**D**) The level of active ERK (P-ERK) were analyzed by Western blot in cells transfected with pre-miR-125b or the control pre-miR after four days of differentiation—the experiment shown in the Figure is representative of two independent experiments; (**E**) The level of the epiblast marker, Fgf5, was measured by qPCR in undifferentiated ESCs and during differentiation upon pre-miR transfection. The epiblast markers, Cerberus and Dnmt3b, were measured at four days of SFEB differentiation in the cells transfected with the indicated pre-miR. The fold change is calculated as indicated in (**C**); (**F**) ChIP-qPCR analysis was performed on chromatin from ESCs transfected with the indicated pre-miR and induced differentiation for four days as SFEBs. The graphs show the methylation state of histone H3 on the promoters of pluripotency (Nanog and Klf2) and epiblast (Fgf5) markers. Data are expressed as fold enrichment relative to the control; (**G**) Immunodeficient mice were injected with ESCs transfected with the pre-miR-125b (right side) and ctrl pre-miR (left side) after three days of differentiation *in vitro* (left panel). Teratomas generated by ESCs overexpressing miR-125b were explanted after one month, and the tissues were analyzed after eosin-hematoxylin staining (right panels) (*p < 0.05).

We screened for miRNAs differentially regulated during ESC neural differentiation and found many miRNAs that are specifically expressed in undifferentiated or differentiated cells [22]. Among these miRNAs, we found that miR-125b is expressed at low levels in ESCs, but its expression increases during the first steps of differentiation. At later time points, miR-125b expression reaches a higher level in differentiated cells (Figure 1A), and in agreement, it is highly expressed in many adult mouse tissues (Figure 1B). We have previously demonstrated that miR-125b, as well as miR-125a, are able to regulate the expression of the BMP4 co-receptor, Dies1 [8], in ESCs, but we still don’t know whether miR-125b may have a role in the early phases of ESC differentiation, when BMP4 regulates the differentiation fate of these cells. The analysis of undifferentiated markers clearly indicated that miR-125b overexpression (Figure S1) does not impair the undifferentiated state of ESCs (Figure 2A); thus, we explored whether miR-125b affects the differentiation program. ESCs transfected with pre-miR-125b were induced to differentiate as serum-free embryoid bodies (SFEBs), which mainly give rise to neuroectoderm derivatives at four days of differentiation. We found that the overexpression of miR-125b blocks ESC differentiation. Indeed, we found a decrease of neuroectodermal markers, whereas the expression of stemness markers is maintained at a high level (Figure 2B,C and Figure S2). These effects are accompanied by an impairment of ERK activation that suggests the failure of proper differentiation (Figure 2D). Considering that SFEB differentiation favors the transition through the epiblast stage that then leads to the formation of neuroectoderm, we performed an analysis to see if the block of differentiation observed upon miR-125b overexpression occurs before or after the epiblast transition. We found that the epiblast markers, Fgf5, Cerberus and Dnmt3b (Figure 2E), were significantly high at four days of differentiation, indicating that the cells are blocked in the epiblast stage. To verify that miR-125b overexpression is able to maintain the epiblast stem cell (EpiSC) phenotype, rather than simply slowing down the differentiation, we analyzed the methylation state of epiblast marker genes. We found that differentiated cells at day 4 upon miR-125b overexpression had epigenetic markers similar to those of EpiSCs (Figure 2F), thus suggesting, again, that these cells may still be pluripotent. To verify this hypothesis, we decided to test the pluripotency of this epiblast cell obtained upon miR-125b overexpression *in vivo*. Thus, we injected, into immunodeficient mice, cells transfected with miR-125b or with a control miR and pre-differentiated *in vitro* for three days. We found that miR-125b overexpressing cells differentiated for three days are still able to form an extensive differentiated teratoma (Figure 2G) in four out of the five mice injected with the cells. The control cells induced the formation of a small, not completely differentiated tumor (data not shown) only in one mouse over five injected. These results demonstrated that miR-125b overexpression is able to sustain the undifferentiated phenotype, even three days after the induction of differentiation and that a large fraction of these cells maintains the pluripotency.

### 2.2. miR-125b Effects on the ESC-Epiblast Transition Are Due to Dies1

**Figure 3:**
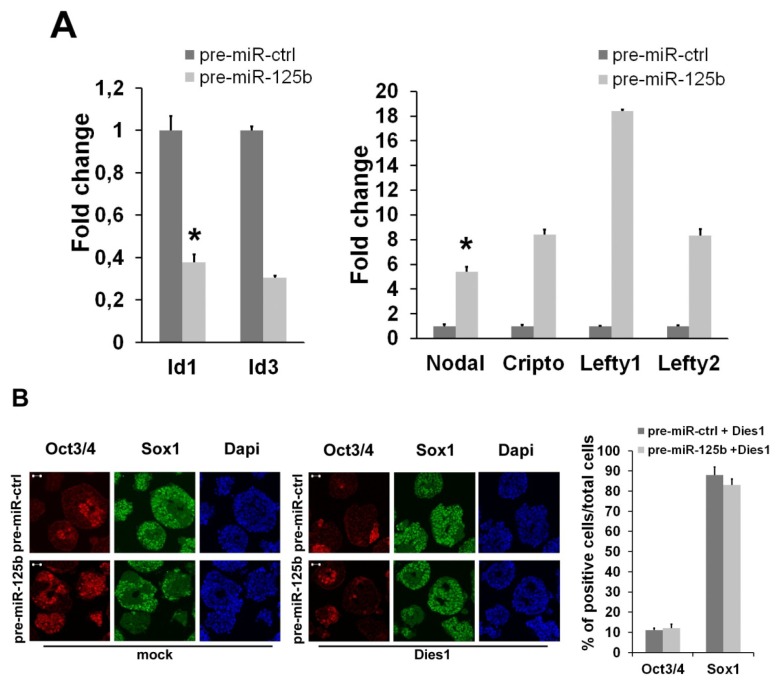

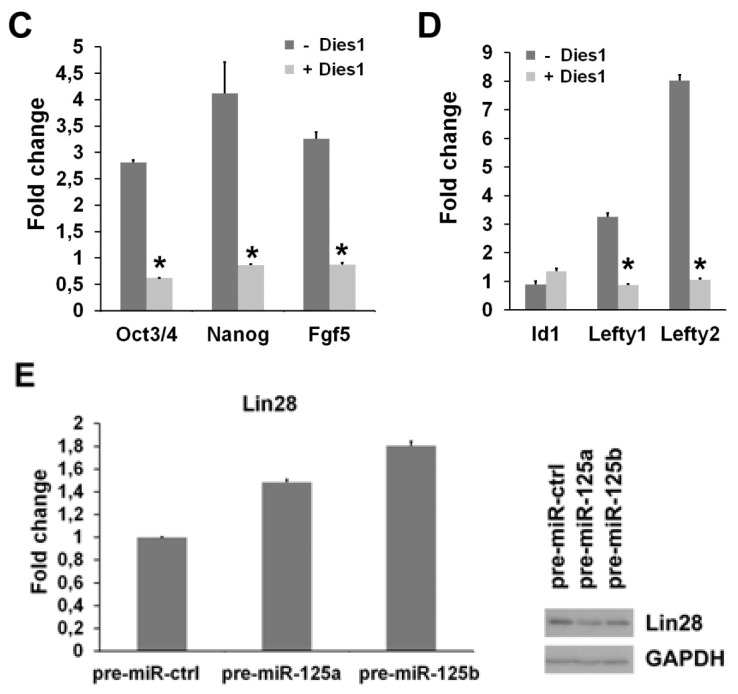
The effects of miR-125b overexpression on TGFβ signaling are mediated by the BMP4 co-receptor, Dies1. (**A**) qPCR analysis of the expression levels of BMP4 (Id1, Id3) and Nodal/Activin (Nodal, Cripto, Lefty1, Lefty2) target genes upon miR-125b overexpression; (**B**) Analysis of the phenotype of ESCs co-transfected with the indicated pre-miR and with the vector expressing Dies1 lacking its 3′UTR or with the empty vector (mock). The expression of stemness (Oct3/4) and neuroectodermal (Sox1) markers was analyzed by immunostaining in cells differentiated as SFEBs for four days. Scale bar: 20 μm; (**C**) q-PCR analysis of the effects of Dies1 re-expression in ESCs transfected with the indicated pre-miR. After four days of differentiation, the expression of stemness (Oct3/4, Nanog) and epiblast (Fgf5) markers was analyzed; (**D**) q-PCR analysis of the expression of BMP4 (Id1) and Nodal/Activin (Lefty1 and Lefty2) targets in four-day differentiated ESCs re-expressing, or not, Dies1 upon miR-125b overexpression. Data in (**C**) and (**D**) are shown as fold changes relative to cognate controls; (**E**) The effects of miR-125a and b overexpression on Lin28 level were analyzed in four-day differentiated SFEBs by means of q-PCR (left panel) and Western blot (right panel). Data are expressed as fold change relative to the control (*p < 0.05).

Based on the evidence that miR-125b is able to control the expression of Dies1 [8], the BMP4 co-receptor, we analyzed whether miR-125b overexpression alters the signaling of BMP4. miR-125b overexpression induced a significant decrease of BMP4 targets during ESC differentiation, accompanied by an evident increases of Nodal/Activin targets (Figure 3A). To address whether these effects of miR-125b overexpression are due to the suppression of Dies1, we tried to rescue the proper differentiation and the proper balance between BMP4 and Nodal pathways by re-expressing a form of Dies1 insensitive to the miR-125b. We found that Dies1 is able to fully rescue the block at the epiblast stage induced by miR-125b (Figure 3B,C). Moreover, this rescue corresponds to the restoration of the proper expression levels of BMP4 and Nodal/Activin targets (Figure 3D). A recent paper has indicated that miR-125b targets Lin28 in ESCs to regulate mesendodermal differentiation [23]. To explore this point, we analyzed the mRNA and protein levels of Lin28 upon miR-125b overexpression during SFEB differentiation. Interestingly, we found that Lin28 expression is not impaired in this context (Figure 3E), indicating that the block at the epiblast stage induced by miR-125b overexpression is not due to the repression of Lin28. Instead, we found that miR-125a overexpression induces a slight decrease of Lin28 protein level, suggesting that these two miRNAs can act during the early phases of ESC differentiation by regulating different subsets of targets.

### 2.3. miR-125 Suppression Promotes ESC Differentiation

**Figure 4:**
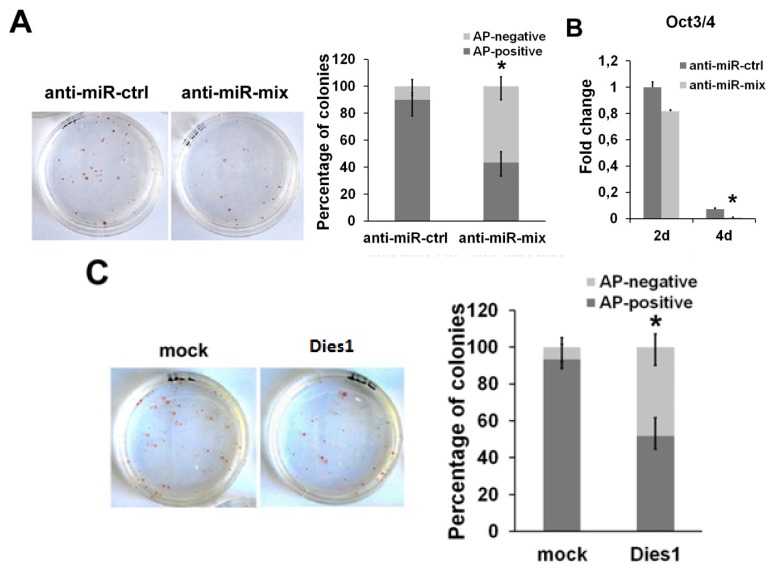
mir-125 suppression induces ESC differentiation. (**A**) Alkaline phosphatase (AP) staining was performed on cells transfected with the mix of anti-miR-125a and b (anti-miR-mix) and with the control anti-miR (anti-miR-ctrl) and cultured for seven days at clonal density in the presence of Leukemia Inhibitory Factor (LIF). The histogram represents the number of AP-positive and -negative colonies; (**B**) q-PCR analysis showing the expression of Oct3/4 in differentiating ESCs upon suppression of miR-125a and b. Data are expressed as fold change relative to the control; (**C**) AP staining of ESCs overexpressing Dies1 after seven days of culture at clonal density in the presence of LIF. The number of AP-positive and -negative colonies is reported in the graph (*p < 0.05).

Considering the evident phenotype induced by miR-125b overexpression, we asked if the suppression of this miR may have an effect on ESCs. To analyze the effects of the depletion of miR-125b in ESC differentiation, we suppressed the endogenous miRNAs by transfecting a mix of anti-miR-125a and anti-miR-125b to avoid the levels of endogenous miR-125a from being able to be substituted for the absence of miR-125b. An anti-miR with no complementarity to any known miRNAs was used as negative control. After anti-miR transfection, we cultured ESCs at low density, and after seven days, we performed an alkaline phosphatase (AP) staining to assay their ability to maintain an undifferentiated phenotype. As shown in Figure 4A, the cells transfected with anti-miR-125 showed a reduction in the number of AP-positive colonies compared to the control, indicating that miRNA suppression causes the loss of the undifferentiated state, even in the presence of LIF. Moreover, we found a rapid decrease of the stemness marker, Oct3/4, upon miR-125 suppression during differentiation, already at day two of SFEB formation, which became more evident at day four (Figure 4B). To better define the correlation between miR-125b and Dies1 in this context, we analyzed the effects of Dies1 ectopic expression in ESCs in the same conditions used for miRNA suppression. We found that Dies1 forced expression induces the decrease of the number of AP-positive colonies, thus resembling the phenotype observed upon miR-125 suppression (Figure 4C). These results support the idea that the levels of the two miR-125 are functionally correlated to that of Dies1 in ESCs.

### 2.4. miR-125b Is Not Regulated by a TGFβ Signaling

**Figure 5:**
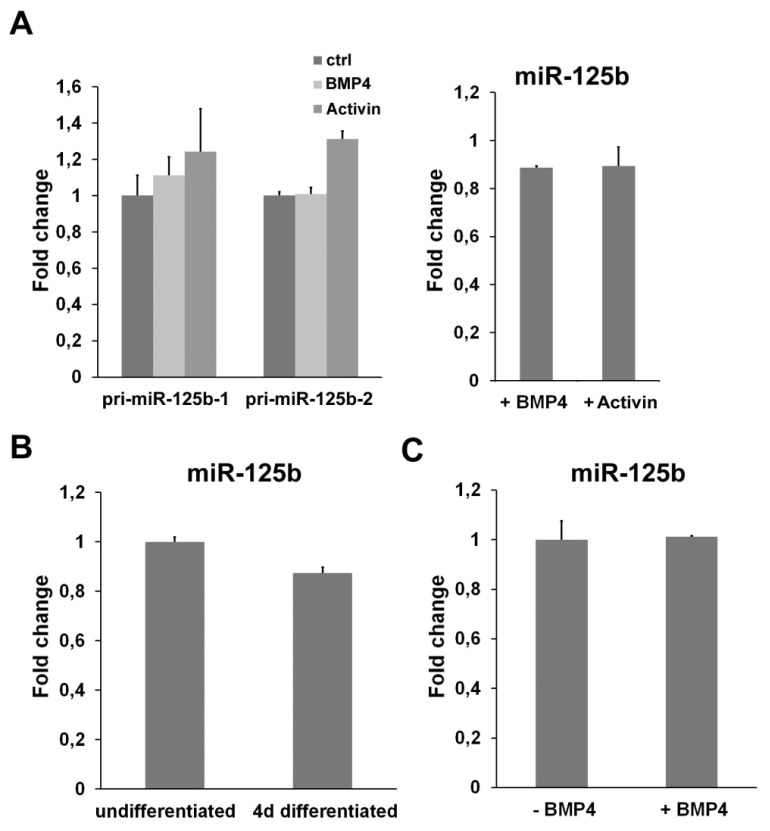
miR-125b expression is independent from TGFβ. (**A**) Analysis of the level of miR-125b after the exposure of ESCs to BMP4 or Activin. The expression of pri-miRs (left panel) was measured after 1 h of treatment with the indicated molecules by using specific primers that distinguish between the transcripts deriving from the two miR-125b genes (pri-miR-125b-1 and pri-miR-125b-2). The level of mature miR-125b was measured after 24 h of BMP4 or Activin treatment; (**B**) Expression level of miR-125b in undifferentiated and four-day differentiated C2C12 cells; (**C**) q-PCR analysis of the expression of miR-125b in C2C12 cells after 24 h of treatment with BMP4. All the data are expressed as fold change relative to the control.

We have shown that miR-125b overexpression impairs both BMP4 and Nodal/Activin pathways. To understand if the miR-125b can be directly regulated by one of these signalings, we exposed ESCs to BMP4 or Activin and analyzed the level of the two pri-miR-125b transcribed from the two miR-125b genes and the mature miR-125b. As shown in Figure 5A, we did not find any significant changes in the expression of miR-125b upon BMP4 or Activin treatment, indicating that this miR is not directly regulated by these two pathways. To verify that this independence of miR-125b from TGFβ is not context-dependent, we analyzed the changes in the possible expression of miR-125b in a different experimental setting. To this aim, we used C2C12, a system in which both miR-125b and BMP4 are able to regulate the differentiation [24,25] and in which miR-125b is highly expressed (Figure 5B). After the exposure of these cells to BMP4, we didn’t find any changes in the expression level of miR-125b (Figure 5C). All these data suggest that miR-125b is able to impair the TGFβ pathway, but is not directly controlled by these molecules.

## 3. Experimental Section

### 3.1. Cell Culture, Transfection and Differentiation

E14Tg2a mouse ESCs (BayGenomics, San Francisco, CA, USA) were maintained on feeder-free, gelatin-coated plates in the following medium: Glasgow Minimum Essential Medium (GMEM, Sigma, St. Louis, MO, USA) supplemented with 2 mM glutamine (Invitrogen, Carlsbad, CA, USA), 1 mM sodium pyruvate (Invitrogen, Carlsbad, CA, USA), 1× nonessential amino acids (Invitrogen, Carlsbad, CA, USA), 0.1 mM β-mercaptoethanol (Sigma, St. Louis, MO, USA), 10% FBS (Hyclone,Watham, MA, USA) and 10^3^ U/mL Leukemia Inhibitory Factor (LIF, Millipore, Billerica, MA, USA). Serum-free embryoid body (SFEB) differentiation was performed by plating 1 × 10^6^ ESCs in 100 mm Petri dishes (BD Biosciences, San Jose, CA, USA) in the following differentiation medium: GMEM supplemented with 2 mM glutamine, 1 mM sodium pyruvate, 1× nonessential amino acids, 0.1 mM β-mercaptoethanol and 10% Knock-out Serum Replacement (KSR, Invitrogen, Carlsbad, CA, USA). In this condition, after 2 days, the cells express a high level of epiblast markers that strongly decrease at 4 days of SFEB differentiation. EpiSCs can be derived with high yield at 2 days of SFEB differentiation, whereas at 3 and 4 days of differentiation, the yield of EpiSC derivation strongly decreases [5].

C2C12 myoblasts were maintained in Dulbecco’s Modified Eagle Medium (Sigma, St. Louis, MO, USA) supplemented with 2 mM glutamine (Invitrogen, Carlsbad, CA, USA), 100 U/mL penicillin/streptomycin (Invitrogen, Carlsbad, CA, USA) and 10% FBS (GIBCO, Life Technologies Italia, Monza, Italy). For differentiation to myotubes, 20 × 10^3^ cells/cm^2^ were plated, and the following day, the medium was substituted with the following differentiation medium: DMEM with 2 mM glutamine (Invitrogen, Carlsbad, CA, USA) and 2% horse serum (Sigma, St. Louis, MO, USA). After 4 days of differentiation, the cells were collected.

Transfection of pre-miRs, anti-miRs (both from Ambion, Austin, TX, USA) and plasmid for Dies1 expression was performed using Lipofectamine 2000 (Invitrogen, Carlsbad, CA, USA), following the manufacturer’s instructions.

### 3.2. Cell Treatment and Alkaline Phosphatase Staining

For cell treatment, ESCs and C2C12 were grown overnight in KSR containing medium with LIF or DMEM plus 1% FBS, respectively and, then, treated for the indicated time with 20 ng/mL of BMP4 or Activin (both from R&D, Minneapolis, MN, USA).

Alkaline phosphatase staining was performed culturing ESCs at clonal density (20–50 cells/cm^2^). After 7 days, the cells were fixed in 10% cold Neutral Formalin Buffer (10% formalin, 110 mM Na_2_HPO_4_, 30 mM NaH_2_PO_4_ in H_2_O) for 15 min and, then, rinsed in distilled water for 15 min. The staining was obtained by incubation for 45 min at room temperature with the following staining solution: 0.1 M Tris-HCl, 0.01% naphthol AS MX-PO4 (Sigma, St. Louis, MO, USA), 0.4% *N*,*N*-dimethylformamide (Sigma, St. Louis, MO, USA) and 0.06% Red Violet LB salt (Sigma, St. Louis, MO, USA).

### 3.3. RNA Isolation, q-PCR and TaqMan Analysis

Total RNA from ESCs and C2C12 was extracted by using TRI-Reagent (Sigma, St. Louis, MO, USA). For the first-strand cDNA synthesis, the manufacturer’s instructions (M-MLV RT, New England BioLabs, Ipswich, MA, USA) were followed. Q-PCR was carried out on an ABI PRISM 7900HT Sequence Detection System (Applied Biosystems, Foster City, CA, USA) using Power SYBR Green PCR Master mix (Applied Biosystems, Foster City, CA, USA). The housekeeping GAPDH mRNA was used to normalize the samples, using the 2^−ΔΔCt^ method. The gene-specific primers used are listed in Table S1.

For the measurement of mature miRNA, RNA was extracted with a mirVana microRNA Isolation kit (Ambion, Austin, TX, USA), according to the manufacturer’s instructions. Single-stranded cDNA was synthetized from 10 ng of total RNA combined with the specific primer for miR-125a or miR-125b or U6 as internal control by using a TaqMan MicroRNA reverse transcription kit (Applied Biosystems, Foster City, CA, USA). miRNA levels were measured by using a TaqMan MicroRNA detection kit (Applied Biosystems, Foster City, CA, USA) with the 7500 Real Time PCR System instrument and the Sequence Detection Systems (SDS) software version 1.4 (Applied Biosystems, Foster City, CA, USA) [26].

### 3.4. Northern Blot Analysis

For Northern blot analysis total RNA was isolated by using the TRI Reagent (Sigma, St. Louis, MO, USA), according to the manufacturer’s instructions. For each sample, 20 μg of total RNA were fractionated on 15% TBE-Urea gel (Criterion precast Gel, Bio-Rad, Segrate, Italy), stained with EtBr (Sigma, St. Louis, MO, USA) for loading control, then transferred to a Hybond membrane (Amersham Pharmacia, Milan, Italy) and, finally, fixed by UV cross-linking in a Stratalinker (Stratagene,La Jolla, CA), according to the manufacturer’s instruction. The membrane was hybridized with 10 pmol of miRCURY LNA Detection Probe, digoxigenin labeled (Exiqon, Vedbaek, Denmark), according to the manufacturer’s instruction. The signal detection was obtained by an anti-digoxigenin-alkaline phosphatase antibody (Roche, Basel, Switzerland) and a chemiluminescent substrate (CDP-Star, Roche, Basel, Switzerland), according to the manufacturer’s instruction.

### 3.5. Protein Extraction and Western Blot Analysis

For protein extracts, cells were lysed in a buffer containing 20 mM Tris-HCl (pH 7.5), 150 mM NaCl, 1 mM EDTA, 1% Triton, 1% sodium deoxycholate and protease inhibitor cocktail (Sigma, St. Louis, MO, USA). The antibodies used for blotting were: Anti-Lin28 (1:700, Abcam,Cambridge, UK); anti-phospho-Erk1 (1:1000, Cell Signaling, Danvers, MA, USA); anti-Erk1 (1:1000, Santa Cruz, Santa Cruz, CA, USA); anti Oct3/4 (1:1000, Santa Cruz, Santa Cruz, CA, USA); and anti-GAPDH (1:1000, Santa Cruz, Santa Cruz, CA, USA).

### 3.6. Immunostaining

For immunostaining, 4-day differentiated SFEBs were fixed in 4% paraformaldehyde and processed, as described in Parisi *et al*., 2012 [12]. The following primary antibodies were used: anti-Oct3/4 (1:200, Santa Cruz, Santa Cruz, CA, USA), anti-Nanog (1:500, Calbiochem, San Diego, CA, USA) and anti-Sox1 (1:100, Santa Cruz, Santa Cruz, CA, USA). The appropriate secondary antibodies were used (1:400, Alexa Molecular Probes, Invitrogen, Carlsbad, CA, USA). Confocal microscopy was performed with an LSM 510 Meta microscope (Zeiss, Milan, Italy) using LSM 510 Meta software [27] and the LSM Image Browser (Zeiss, Milan, Italy). The brightness, contrast and color balance of the images were adjusted in Photoshop CS2 (Adobe Systems, Agrate Brianza, Italy).

### 3.7. Teratoma Formation

ESCs transfected with pre-miR-125b or pre-miR-ctrl were differentiated as SFEBs for 3 days. Then, SFEBs were dissociated, and 2 × 10^6^ cells were used for subcutaneous injection in nude mice. Four weeks after the injection, tumors were surgically dissected from the mice. Samples were fixed in 4% paraformaldehyde and embedded in paraffin. Sections were stained with hematoxylin and eosin.

### 3.8. Chromatin Immunoprecipitation (ChIP)-qPCR Analysis

Chromatin immunoprecipitation was performed as described previously [28]. To immunoprecipitate soluble chromatin extracts, anti-H3K4-3me (Millipore, Billerica, MA, USA) and anti-H3K27-3me (Millipore, Billerica, MA, USA) antibodies were used. Appropriate IgGs were used as negative control. Supernatant obtained without antibody was used as input control. After q-PCR, the amount of precipitated DNA was calculated relative to the total input chromatin and expressed as the percentage of total chromatin, according to the formula 2*^ΔCt^*, where *Ct* represents the cycle threshold and *ΔCt* = *Ct* (input) − *Ct* (immunoprecipitation). The gene-specific primers used are listed in Table S1.

### 3.9. Statistics

Data are presented as the means ± SD of at least three independent experiments. Whenever necessary, the statistical significance of the data was analyzed using the Student’s *t*-test (* *p* < 0.05).

## 4. Conclusions

We demonstrated that miR-125b is essential for the proper differentiation of ESCs, based on the relevant effects of its overexpression and suppression. We have previously demonstrated that miR-125a had similar effects on ESC differentiation [8], thus suggesting that these two miRNAs work cooperatively through the suppression of Dies1, the co-receptor of BMP4 [29]. This relationship is an elegant example of how multiple miRNAs can converge on a single pathway to promote a common outcome. Interestingly, while miR-125a is directly regulated by BMP4, miR-125b seems to not be regulated by TGFβ signaling. This observation suggests that the BMP4 signaling in the first steps of ESC differentiation undergoes different regulations that are dependent (miR-125a) or independent (miR-125b) by itself. This can probably be due to the relevance that the balance between BMP4 and Nodal/Activin pathways have in the control of the transition from ESC to the epiblast stage, thus indicating that ESCs modulate in different ways such important pathways.

## Supplementary Information
