# Supplementary material for: miR-125b Regulates the Early Steps of ESC Differentiation through Dies1 in a TGF-Independent Manner
Source: Int J Mol Sci. 2013 Jun 27;14(7):13482–96. doi: 10.3390/ijms140713482 (PMC3742198; doi:10.3390/ijms140713482)
Supplement: Supplementary file 1 [file ijms-14-13482-s001.pdf]

Supplementary Information

Table S1. Primers used for qPCR.

Name [a]	Forward primer	Reverse primer	Application
Cerberus	ACTGTGCCCTTCAACCAGACCATTG	TGCCCCTTCTCCGGGAAAACGA	RT-qPCR
Cripto	ATCCAGTGTGGTTTTGCTTGTG	TCTCTGATGGCAAGGTCTCTCC	RT-qPCR
Dnmt3b	CCAAGGACACCAGGACGCGC	TCCGAGACCTGGTAGCCGGAA	RT-qPCR
Fgf5	CCTCATCTTCTGCAGCCACCTGATC	GTTCCGAGCCGCTTCCTTGGCTGCC	RT-qPCR
Gapdh	GTATGACTCCACTCACGGCAAA	TTCCCATTCTCGGCCTTG	RT-qPCR
Id1	GAGCAGCAGGTGAACGTCCT	TCCTTGAGGCGTGAGTAGCA	RT-qPCR
Id3	GTAAGAGCCCGTCGACCGA	GCAGTGGTTCATGTCGTCCA	RT-qPCR
Klf2	CCAACTGCGGCAAGACCTAC	CAATGATAAGGCTTCTCACCTGTGT	RT-qPCR
Klf5	GGTCCAGACAAGATGTGAAATGG	TTTATGCTCTGAAATTATCGGAACTG	RT-qPCR
Lefty1	CTCGGGTCACCATTGAATGG	TGGACACGAGCCTAGAATCGA	RT-qPCR
Lefty2	GTCACCATTGAATGGCTGAGAG	GTGGATGGACACGAGCCTAGAG	RT-qPCR
Lin28	GTTCGGCTTCCTGTCTATGACC	CTTCCATGTGCAGCTTGCTCT	RT-qPCR
Nanog	TCAGAAGGGCTCAGCACCA	GCGTTCACCAGATAGCCCTG	RT-qPCR
Nodal	CCTCCAGGCGCAAGATGT	ACCAGATCCTCTTCTTGGCTCA	RT-qPCR
Oct3/4	AACCTTCAGGAGATATGCAAATCG	TTCTCAATGCTAGTTCGCTTTCTCT	RT-qPCR
Pax6	AGTGAATGGGCGGAGTTATG	ACTTGGACGGGAACTGACAC	RT-qPCR
pri-miR-125b-1	GAGTCTGCAACCGAAATTGCCTG	GTTCCTTCAGCGATGCAAAGGC	RT-qPCR
pri-miR-125b-2	GCTGTCCGTTTACCTGGAAGAAG	CTGGTGGTTTATGCCGAGAATC	RT-qPCR
Fgf5	AGGGACGGTCAAGATTCCTT	AGAACCAGCAGAGTCCCAGA	ChIP-qPCR
Nanog	TGTGAGCTCAGTGCTCCTTCCAAA	TTCAGACCTTGGCTCCAGATGCTA	ChIP-qPCR
Klf2	TGCAGATCTTGAGGGCCTAGTTGT	TCCCATGGAGAGGATGAAGTCCAA	ChIP-qPCR

[a] The primer name is reported as indicated in the Figures.

Figure S1. miR-125b level upon pre-miR transfection. (**A**) RNA samples prepared from ESCs transfected with the control pre-miR and pre-miR-125b were subjected to Northern blot analysis with a specific probe for miR-125b that reveals the level of pre-miR and mature miR. U6 snRNA was used as a loading control; (**B**) TaqMan microRNA analysis to evaluate the level of miR-125b in ESCs transfected with the control pre-miR and pre-miR-125b. The data are expressed as fold change relative to the control.

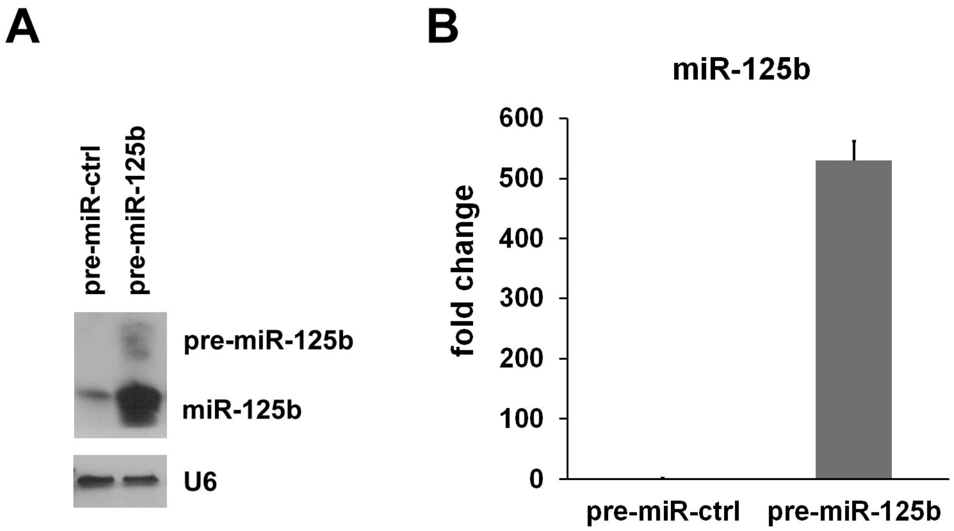

Figure S2. Oct3/4 level in 4 days differentiated ESCs upon pre-miR transfection. The protein levels of Oct3/4 were measured by western blot in ESCs transfected with the indicated pre-miR and differentiated as SFEBs for 4 days .

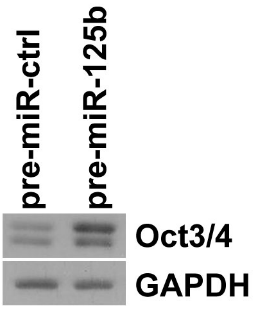

© 2013 by the authors; licensee MDPI, Basel, Switzerland. This article is an open access article distributed under the terms and conditions of the Creative Commons Attribution license (http://creativecommons.org/licenses/by/3.0/).
